# Preventive effect of ulinastatin on postoperative complications, immunosuppression, and recurrence in esophagectomy patients

**DOI:** 10.1186/1477-7819-11-84

**Published:** 2013-04-10

**Authors:** Lingmin Zhang, Ning Wang, Suna Zhou, Wenguang Ye, Qinglin Yao, Guixia Jing, Mingxin Zhang

**Affiliations:** 1 Department of Anesthesiology, First Affiliated Hospital, Medical School, Xi’an Jiaotong University, Xi’an 710061, Shaanxi Province, China; 2 Department of Anesthesiology, Second Affiliated Hospital, Medical School, Xi’an Jiaotong University, Xi’an 710061, Shaanxi Province, China; 3 Department of Radiotherapy, Tangdu Hospital, Fourth Military Medical University, Xi’an 710038, Shaanxi Province, China; 4 Department of Gastroenterology, Tangdu Hospital, Fourth Military Medical University, Xi’an 710038, Shaanxi Province, China

**Keywords:** Esophagectomy, Immunosuppression, Postoperative complications, Recurrence, Ulinastatin

## Abstract

**Background:**

To evaluate the potential efficacy of preventive effect of ulinastatin in esophagectomy patients.

**Methods:**

Eighty patients with esophageal cancer were preoperatively allocated at random into two equal groups. Ulinastatin was administered to the treatment group (U) whereas the control group (C) received a placebo. The arterial oxygen tension and carbon dioxide tension were measured and the respiratory index (RI) was calculated. Plasma levels of circulating T lymphocyte subsets and interleukin 6 (IL-6) were measured and clinical courses of patients in the two groups were compared.

**Results:**

RI in the U group was significantly lower than that in the C group. The rate of postoperative complications and the duration of ICU stay were significantly lower in the U group. Ulinastatin significantly increased the rate of CD3^+^ and CD4^+^ cells, and ratio of CD4^+^/CD8^+^, but decreased the rate of CD8^+^ cells and release of IL-6 compared to the C group on postoperative days 1 and 3. Patients within the C group showed worse recurrence free survival. Multivariate analysis revealed that ulinastatin administration significantly decreased the incidence of recurrence.

**Conclusions:**

Ulinastatin had a preventive effect on postoperative complications and immunosuppression in esophagectomy patients, thereby prolongingrecurrence free survival.

## Background

Surgery remains the most effective treatment for solid tumors including esophageal cancer. However, esophagectomy, one of the most invasive procedures among gastrointestinal operations, has a high frequency of postoperative complications [1]. Several responsible background factors have been proposed to explain the broad spectrum of postoperative complications after such invasive procedures. The most important ones are systemic inflammatory response syndrome and compensatory anti-inflammatory cytokine response syndrome [2-4]. Moreover, surgical stress can cause immunosuppression in response to the complex interaction of various hormones, cytokines, and acute phase reactants [5]. It has been reported that perioperative and postoperative immunosuppression increases the ratio of recurrence and adversely affects the prognosis of cancer patients [6,7]. Therefore, it is desirable to find an effective countermeasure against the overproduction of proinflammatory cytokines, postoperative complications, and immunosuppression.

Ulinastatin is a serine protease inhibitor with a molecular weight of ~67,000 found in healthy human urine. It is used worldwide for patients with inflammatory disorders, including disseminated intravascular coagulation, shock, and pancreatitis [8-10]. Furthermore, ulinastatin administration can help reduce the surgical stress, prevent radiation-induced lung injury, and modulate immune functions [11-13].

The aim of the present study was to evaluate the potential efficacy of preventive effect of ulinastatin on postoperative complications, immunosuppression, and recurrence in esophagectomy patients.

## Methods

### Patients

Between January 2007 and December 2007, patients with lower thoracic esophageal cancer requiring surgical intervention at the First Affiliated Hospital and Second Affiliated Hospital, Medical College of Xi’an Jiaotong University, were enrolled. Exclusion criteria: prior chemotherapy or irradiation or immunosuppressive drug administration; blood loss ≥ 1,000 mL; ASA classification ≥ , histological type of adenocarcinoma. Eighty patients were subsequently randomized into two groups: control group (C, n = 40) and ulinastatin group (U, n = 40). The operative procedure for removal of the cancer was performed by a single surgical team and through the left posterolateral thoracotomy approach with combined thoracoabdominal lymphatic dissection, proximal gastric resection and mobilization of the stomach for esophageal replacement. Institutional Ethics Committee approval for this project was obtained. Written informed consent was obtained from each patient before randomization. The study was designed as a single blinded study. Ulinastatin (Miraclid, Mochida Pharmaceulinastatincal, Japan) was administered to the U group as a bolus of 200,000 U diluted in 20 mL of normal saline every 24 h from 3 days pre-operation until 3 days post-operation.

### Clinical course evaluation

Clinical course was evaluated based on rate of postoperative complications, including cardiovascular complications (arrhythmia, pulmonary embolism, and myocardial infarction), pulmonary complications (pneumonia, atelectasis, pulmonary edema), and others (esophagogastric anastomosis leakage, stenosis, and wound infection). The criteria of postoperative complications, especially for pulmonary complications, were described as before [14]. The duration of ICU and hospital stay was also determined. All patients received cisplatin-based postoperative adjuvant chemotherapy or standard radiotherapy, if required. The follow-up period ranged from 1 to 48 months (median, 35.7 months). Computed tomography (CT) was performed at least every 6 months to detect recurrence.

### Sample collection and assay

Arterial blood was collected immediately at 10 minutes after operation began (T_1_), 1 hour after one-lung ventilation (T_2_), and at the time of closure (T_3_). Arterial oxygen tension (Pa_O2_) and carbon dioxide tension (Pa_CO2_) were measured by blood gas analysis. The respiratory index (RI) was calculated as a marker of lung damage using the following formulas: *RI* = [*F*_*IO*2_ × (760–47) - *Pa*_*CO*2_/0.8]/*Pa*_*O*2_. Peripheral whole blood samples were obtained 1 hour before surgery (D_0_) and on postoperative days 1, 3, and 7 (D_1_, D_2_, and D_3_). Lymphocyte subsets were counted by a FACSCalibur (Becton Dickinson, San Jose, CA, USA) flow cytometer. Cytokine levels (IL-6) were determined by ELISA, using commercially available kits (R&D Systems, Minneapolis, MN, USA).

### Statistics

Data are expressed as mean ± standard deviation. Statistical analysis was performed with the SPSS software package (version 13.0, SPSS Institute). Continuous variables were analyzed using repeated measures ANOVA and categorical data were compared by the χ^2^ test or Fisher’s exact test. Survival curves were estimated by the Kaplan-Meier method with the log-rank test. Multivariate analysis was performed using the Cox proportional hazard regression analysis. *P* values < 0.05 were considered significant.

## Results

### Baseline characteristics of enrolled patients

During a period of 12 months between January 2007 and December 2007, 80 patients undergoing esophagectomy were enrolled in this study. Background factors for esophageal cancer patients are listed in Table 1. There were no significant differences between the groups in average age, gender, TNM stage, length of resection, number of lymph node dissection, alcohol consumption, smoking, ASA classification, duration of operation, duration of anesthesia, and blood loss during operation. Type of anesthesia was the same between the two groups. There were also no significant differences in perioperative management, including the usage of steroid and elastase inhibitor, infusion and nutritional support, and NSAIDs and other analgesics, between the two groups.

**Table 1:** Baseline characteristics of the 80 patients

	**Control (n = 40)**	**Ulinastatin (n = 40)**	***P***
Age	56 ± 12	56 ± 10	0.861
Gender(male/female)	34/6	33/7	0.762
TNM stage(I/II/III)	7/18/15	6/20/14	0.897
Length of resection (cm)	10 ± 4.8	11 ± 4.0	0.157
Number of lymph node dissection	11 ± 4.1	10 ± 4.6	0.154
Alcohol consumption (yes/no)	25/15	23/17	0.648
Smoker(yes/no)	21/19	20/20	0.823
FEV1/FVC(%)	85.3 ± 3.3	85.1 ± 4.3	0.769
ASA classification (I/II)	18/22	17/23	0.822
Duration of operation (min)	206 ± 44	207 ± 43	0.918
Duration of anesthesia (min)	240 ± 46	242 ± 44	0.862
Blood loss during operation (mL)	520 ± 43	518 ± 62	0.903

### Effect of ulinastatin on respiratory index

**Figure 1:**
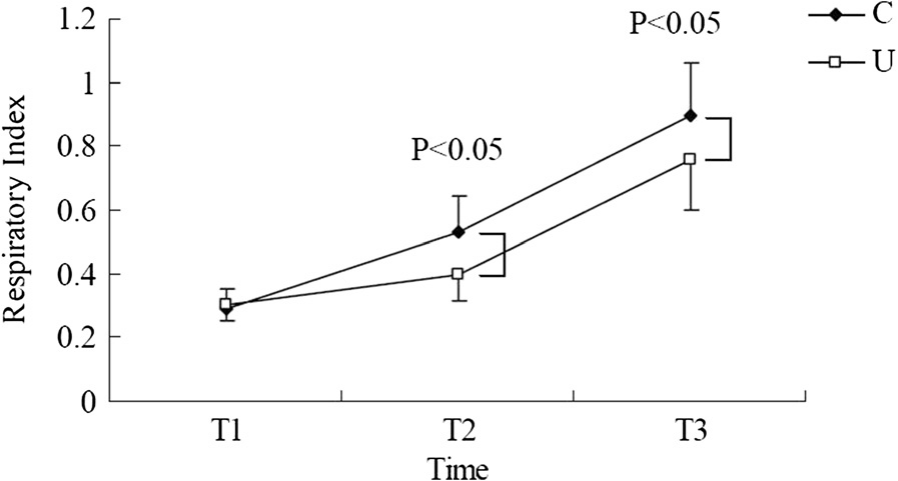
**Effect of ulinastatin on respiratory index.** Respiratory index (RI) in the ulinastatin group (U) was significantly lower than that in the control group (C) 1 hour after one-lung ventilation (T_2_) and the time of sternal closure (T_3_; *P* < 0.05). The RI was calculated as a marker of lung damage using the following formulas: *RI* = [*F*_*IO*2_ × (760–47) - *Pa*_*CO*2_/0.8]/*Pa*_*O*2_.

RI before operation did not differ significantly between the groups (group U *vs.* C, 0.29 ± 0.07 *vs.* 0.31 ± 0.06), and there were significant time-dependent changes in RI value in both groups (*P* < 0.05, Figure 1). Group U showed significantly lower RI values than that of group C, both at 1 hour after one-lung ventilation (T_2_) (0.40 ± 0.09 *vs.* 0.53 ± 0.11, *P* < 0.05) and the time of sternal closure (T_3_) (0.75 ± 0.16 *vs.* 0.90 ± 0.17, *P* < 0.05).

### Effect of ulinastatin on postoperative clinical course

The postoperative clinical course of each patient was carefully monitored daily, and complications were checked (Table 2). Postoperative complications were observed in 12 patients (30%) in the C group and 4 patients (10%) in the U group, respectively (*P* < 0.05). A significant decrease in pulmonary complications was observed in the U group (*P* < 0.05), and one patient in the C group died of pulmonary oedema. Although length of hospital stay showed no significant differences between the two groups, the duration of ICU stay was significantly shorter in the U group (*P* < 0.05).

**Table 2:** Effect of ulinastatin on postoperative clinical course

	**Control (n = 40)**	**Ulinastatin (n = 40)**	***P***
Cardiovascular complications	1	1	1
Pulmonary complications	8	1	0.034
Anastomosis leakage	1	1	1
Anastomosis stenosis	1	0	1
Wound infection	1	1	1
Total	12	4	0.034
Death	1	0	1
Duration of ICU stay (hours)	45 ± 24	33 ± 16	0.01
Length of hospital stay (days)	11 ± 4	10 ± 2	0.170

**Figure 2:**
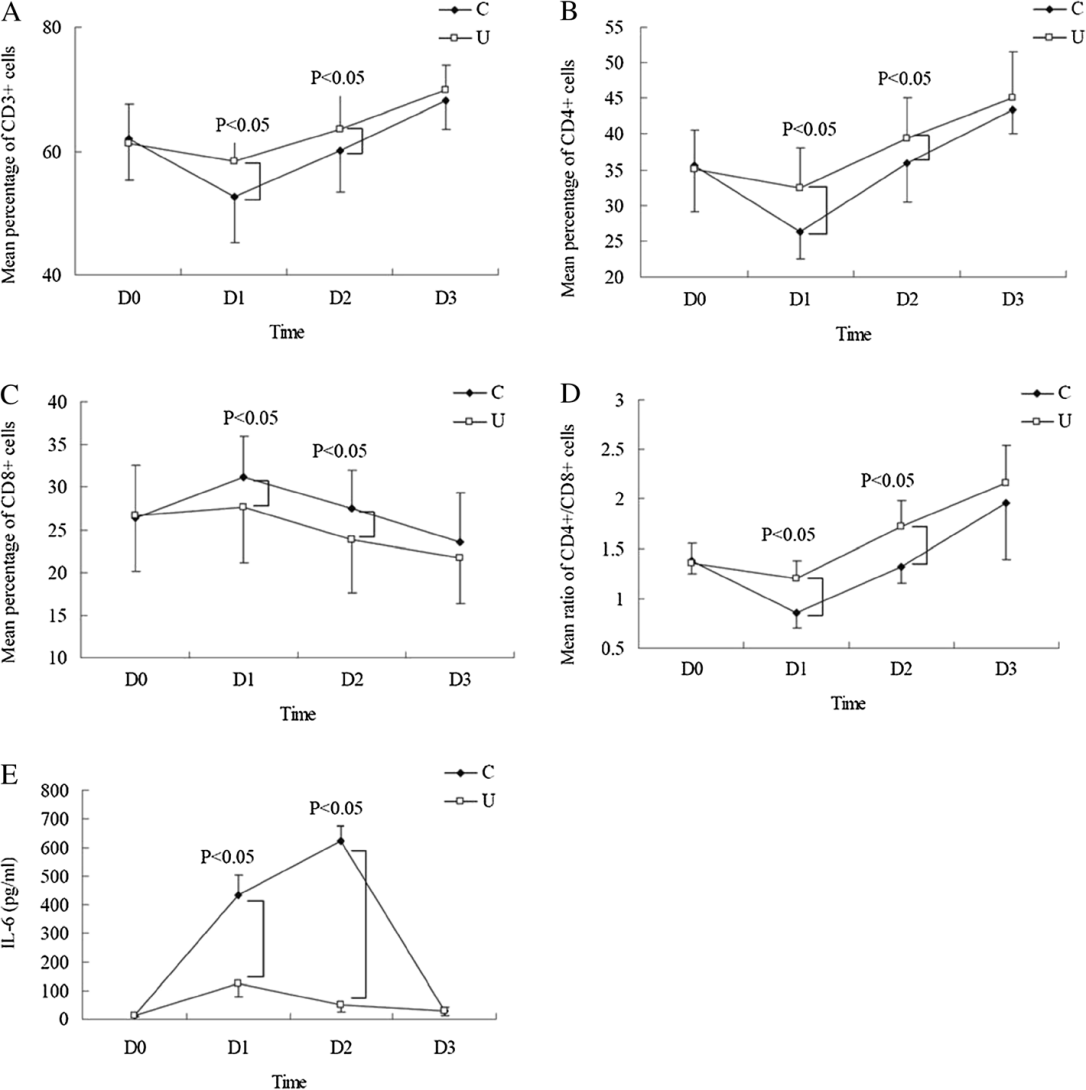
**Effect of ulinastatin on lymphocyte subsets and IL-6.** Ulinastatin (U) administration significantly increased the rate of CD3^+^ (**A**) and CD4^+^ (**B**) cells, and ratio of CD4^+^/CD8^+^ (**D**), but decreased the rate of CD8^+^ (**C**) cells and release of IL-6 (**E**) compared to control group (**C**) on postoperative days 1 (D_1_) and 3 (D_2_; *P* < 0.05). D_0_ = 1 hour before surgery, D_3_ = Postoperative day 7.

As can be seen from Figure 2, ulinastatin administration significantly increased the rate of CD3^+^ and CD4^+^ cells, and ratio of CD4^+^/CD8^+^, but decreased the rate of CD8^+^ cells and release of IL-6 compared to the C group on D_1_ and D_2_ (*P* < 0.05).

### Side effects

No patient experienced side effects related to ulinastatin administration; namely, shock, itching, rash, nausea, vomiting, or neutropenia.

### Survival analysis

**Figure 3:**
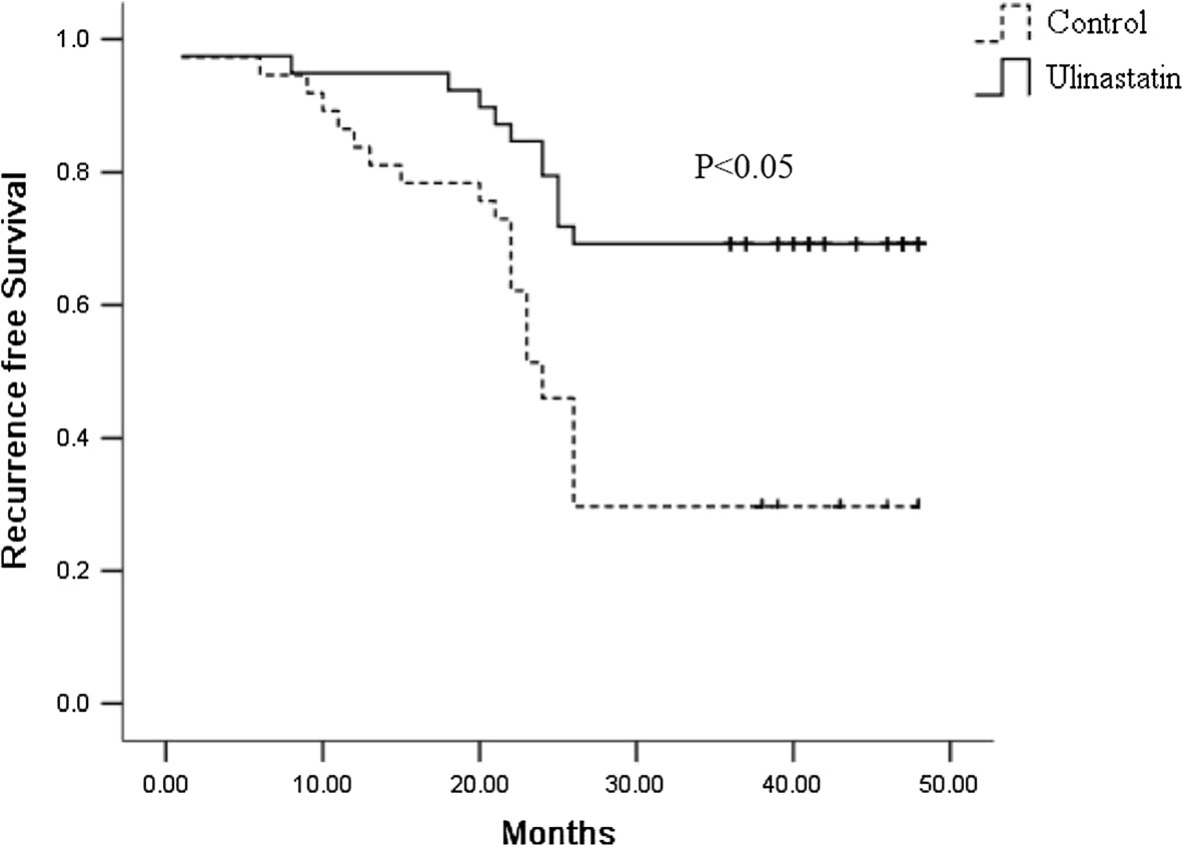
**Kaplan-Meier survival analysis.** Cumulative recurrence free survival differences between patients in the C and U groups. Patients within the C group showed worse recurrence free survival. *P* value was obtained using the log-rank test of the difference.

Of 80 patients in the database, one patient died in the C group during the perioperative period, and 3 were lost to follow-up. As a result, 76 patients were enrolled for survival analysis. The recurrence rate of the U group was 57.5% compared to 72.5% in the C group. The most common recurrence pattern was locoregional recurrence (60% in the U group and 72% in the C group), while other patients developed systemic recurrence or a combination of both. Recurrence-free survival of all patients was 33.8 ± 1.7 months, and it was statistically better for the U group (39.4 ± 2.2) compared to the C group (27.8 ± 2.4) by Kaplan-Meier analysis (*P* < 0.05, Figure 3). Multivariate analysis revealed that ulinastatin administration significantly decreased the incidence of recurrence (Table 3).

**Table 3:** Multivariate cox proportional hazards analysis for recurrence free survival

**Variables**	**Recurrence free survival**		***P***
	RR	95% CI	
Ulinastatin administration	0.149	0.063-0.351	<0.05
TNM	1.812	0.652-5.038	0.254
Alcohol consumption	2.066	0.909-4.344	0.757
Smoking	1.088	0.534-2.217	0.817
Gender	0.916	0.425-1.973	0.822

## Discussion

Major stressful surgery including esophagectomy always caused overproduction of proinflammatory cytokines. The initial proinflammatory response may be uncontrolled causing an imbalance between inflammatory response syndrome and compensatory anti-inflammatory cytokine response syndrome, which led to postoperative complications [15]. For the special surgical procedures, the risk of pulmonary complications after esophagectomy is higher than any other common operation [16]. Moreover, surgical stress can cause immunosuppression in response to overproduction of proinflammatory cytokines. In esophageal cancer, a prognostic relation between the presence of complications and immunosuppression after esophagectomy and survival has previously been reported [17,18]. These data suggest that an effective countermeasure against postoperative complications and immunosuppression is desirable.

Ulinastatin has many physiological effects in surgical stress, including the decrease of the inflammatory reaction, inhibition of immunosuppression, and modification of the water balance [13,19,20]. Moreover, previous studies have shown that ulinastatin inhibits human ovarian cancer and the effect could be related to down-regulation of protein kinase C [21]. Studies have also found that ulinastatin enhances the inhibitory effect of docetaxel in breast cancer by a mechanism consistent with the down-regulated expression of IL-6, IL-8, and TNF-α [22]. Since ulinastatin had a preventive effect on postoperative complications and immunosuppression, and might inhibit the growth of cancer cells, we chose it for the certain purpose.

CD3^+^, CD4^+^, CD8^+^ T-lymphocyte percentage and CD4^+^/CD8^+^ ratio were closely related to the cellular immune function and postoperative anti-tumor immunity [23-25]. Moreover, lower CD3^+^, lower CD4^+^ and lower CD4^+^/CD8^+^ ratio were factors independently associated with worse prognosis of esophageal cancer patients in different reports [26,27]. Therefore, we investigated effect of ulinastatin administration on content of lymphocyte subsets.

In the present study, it was found that ulinastatin administration had a protective effect on pulmonary function by decreasing the increasing trend of RI during operation. As a result, the postoperative complications were lower than that in the C group, especially for pulmonary complications. Low occurrence of postoperative complications shortens the duration of ICU stay and decreased cost of care. Further, we investigated the effect of ulinastatin on release of IL-6 and content of lymphocyte subsets. The change of post-operative IL-6 and lymphocyte subsets reflected beneficial effects of ulinastatin on anti-inflammatory action, postoperative immunosuppression, and postoperative anti-tumor response. Finally, we observed that the U group had a longer recurrence free survival.

## Conclusions

From these results we concluded that ulinastatin had a preventive effect on postoperative complications and immunosuppression in esophagectomy patients,thereby, prolonging recurrence free survival. The possible reason may be that the enhanced anti-tumor response inhibited tumor metastasis [28,29]. However,the detailed mechanism of action of ulinastatin should be further studied at the molecular biological level. Evaluation of a large number of cases is also necessary to assess the clinical usefulness of ulinastatin.

## Abbreviations

IL-6: Interleukin-6; RI: Respiratory index.

## Competing interests

The authors declare that they have no competing interests.

## Authors’ contributions

LM and NW participated in the design and conduction of experiments, data analysis, and final drafting and writing of the manuscript. LM, NW, SZ and WY all contributed to these experiments. GJ and MZ were closely involved in research design and drafting of the final manuscript. All authors read and approved the final manuscript.
